# Human Papillomavirus Prevalence and Genotype Distribution in Unvaccinated Women: A Gynaecology and Obstetrics Hospital-Based Retrospective Study in México

**DOI:** 10.3390/microorganisms14102210

**Published:** 2026-10-01

**Authors:** Saúl Flores-Medina, Selene Sam-Soto, Noemí Guadalupe Plazola-Camacho, Ricardo Figueroa-Damián, Edgar Reyna-Rosas, Cristina Arteaga-Gómez, Leticia Rocha-Zavaleta

**Affiliations:** 1 Departamento de Infectología e Inmunología, Instituto Nacional de Perinatología, Ciudad de México 11000, Mexico; noemi.plazola@inper.gob.mx (N.G.P.-C.); rfd6102@yahoo.com.mx (R.F.-D.); 2 CECyT No. 15 “DAE”, Instituto Politécnico Nacional, Ciudad de México 12100, Mexico; 3 Departamento de Ginecología Quirúrgica, Instituto Nacional de Perinatología, Ciudad de México 11000, Mexico; samsotoselene@gmail.com; 4 Departamento de Biología Molecular y Biotecnología, Instituto de Investigaciones Biomédicas, Universidad Nacional Autónoma de México, Ciudad de México 04510, Mexico; ereyna5@gmail.com; 5 Dirección General, Instituto Nacional de Perinatología, Ciudad de México 11000, Mexico; cristina.arteaga@inper.gob.mx

**Keywords:** human papillomavirus genotypes, unvaccinated women, condyloma acuminatum, cervical ectopy, uterine myomatosis, LSIL, HSIL, cervical cancer, infertility, high-risk pregnancy, menopause

## Abstract

In countries with suboptimal vaccination coverage, human papillomavirus (HPV) genital infections remain a public health problem. Furthermore, the prevalence of specific HPV genotypes in unvaccinated women is uncertain. Therefore, this study aimed to retrospectively evaluate HPV prevalence and genotype distribution among unvaccinated women attending a gynaecology and obstetrics hospital in Mexico, and to establish potential associations between HPV infection and the different pathologies affecting unvaccinated women. A single-centre, retrospective analysis of 347 unvaccinated women who underwent gynaecological examination and had an HPV test was conducted. HPV genotypes in cervical cells were determined using the CLART HPV4 system, which can detect 35 HPV genotypes. Overall, 124 (35.73%) women tested positive for HPV, which included single- and multiple-genotype infections. Oncogenic, high-risk HPV (HR-HPV) genotypes were detected in 74.19% of the HPV-positive women. Ranked by prevalence, HPV59 was the most frequent, followed by HPV16, HPV39, HPV31, and HPV42. Out of the 11 HR-HPV genotypes identified, 6 are targeted by the nonavalent HPV vaccine. In conclusion, this study reports the prevalence of HPV infection among unvaccinated women attending a gynaecological and obstetric centre and demonstrates that HR-HPV genotypes are the most prevalent in this population. These data may facilitate the design of organised screening and vaccination programmes to reduce genital infection and cervical cancer burden in México.

## 1. Introduction

Human papillomavirus (HPV) infection is one of the most common sexually transmitted diseases worldwide. HPVs are non-enveloped, double-stranded DNA viruses, with circular genomes of 5.7–8.7 kbp. According to the International Agency for Research on Cancer (IARC) carcinogen classification, twelve HPV genotypes are included in Group 1 (carcinogenic to humans) and are thus known as high-risk HPV (HR-HPV) genotypes: HPV16, 18, 31, 33, 35, 39, 45, 51, 52, 56, 58 and 59. HPV68 is included in Group 2A (probably carcinogenic to humans), and HPV26, 69, 73, and 82 are part of Group 2B (possibly carcinogenic to humans) [1]; in this work, genotypes from both of these groups will be referred to as pHR-HPV. Interestingly, a systematic analysis showed that 17 HPV genotypes are associated with cervical cancer [2]. The authors reported that HPV16 and 18 account for 75% of cervical cancer cases; HPV31, 33, 45, 52, and 58 contribute 15–20% of cases; while HPV35, 59, 39, 56, 51, 68, 73, 26, 69, and 82 together are associated with approximately 5% of cases globally. In addition, meta-analyses have shown that HPV68, 26, 66, 67, 73, and 82 are more commonly detected in cervical cancer tissue than in normal cervical cells [3]. Finally, other HPV genotypes, primarily HPV6 and 11, have been recognised as etiological agents for anogenital warts; thus, they are considered low-risk genotypes (LR-HPV).

Cervical cancer represents about 80% of all new cancer cases attributable to HR-HPV [4]. Such HR-HPV-associated cervical cancer can be prevented through organised prevention programmes, including cervical screening for detecting precancerous lesions, and HPV vaccination. Intervention based on Pap smears of asymptomatic women is effective in detecting early HPV infections and precancerous lesions. On the other hand, HPV vaccination, either with the quadrivalent Gardasil^TM^ (HPV6, 11, 16, and 18) or the nonavalent Gardasil^®^9 (HPV6, 11, 16, 18, 31, 33, 45, 52, and 58) vaccines, has been demonstrated to be a critical prophylactic approach in preventing HPV infection and further development of cervical neoplasia [5]. Although HPV vaccination was introduced in Latin American and Caribbean countries in 2008, the mean national coverage was only 61% by 2020 [6]. Projections based on statistical data suggested that these countries might reach 80% full HPV coverage by 2030 [6]. However, the drop in vaccination rates during the SARS-CoV-2 pandemic has made achieving higher coverage even more challenging [7].

The effectiveness of HPV vaccination in preventing high-grade cervical lesions [8] and cervical cancer [5] has been demonstrated. Unfortunately, epidemiological data show that incidence rates of cervical cancer in lower-middle-income and low-income countries are significantly higher compared with those in high- and upper-middle-income countries [4]. Inequalities in cervical cancer burden are associated with coverage of HPV vaccination and cervical screening [9]. The impact of HPV vaccination on the prevalence of HPV genotypes included in the vaccines has been evaluated. For example, meta-analysis data showed that after 6 years of HPV vaccine administration, the prevalence of vaccine genotypes decreased by more than 70% [10]. Surprisingly, a decrease in prevalence was also detected in a proportion (17–49%) of unvaccinated females, suggesting a potential herd immunity effect [8]. HPV vaccination was officially introduced in Mexico in 2009. The quadrivalent vaccine was administered until 2024 and was replaced by the nonavalent vaccine in 2025. Unfortunately, vaccination coverage in Mexico is still low.

HPV prevalence has been studied in diverse populations, from young students [11] to attendees of gynaecology and oncology hospitals [12]. However, studies of HPV infection in unvaccinated women attending gynaecology and obstetrics centres are less common. Analysis of these groups of unvaccinated women may provide valuable information about the circulating HPV genotypes and those more frequently associated with gynaecological diseases.

In the present study, women attending the National Institute of Perinatology (INPer) in Mexico City (Mexico) were analysed. INPer provides health services for women with infertility; high-risk pregnancies; menopause; and pathologies directly affecting the female genital tract, such as uterine myomatosis, cervical ectopy, condyloma acuminatum, squamous intraepithelial lesions (SIL), and cervical cancer. A proportion of women referred to INPer have never been vaccinated, and the prevalence of HPV in these women is unknown. Therefore, the aim of this project was to retrospectively evaluate the prevalence of HPV and to determine the genotype distribution in unvaccinated women receiving care at INPer. We also aimed to establish potential associations between HPV infection and the pathologies treated in these unvaccinated women.

## 2. Materials and Methods

### 2.1. Study Population

This was a single-centre, retrospective study conducted at INPer, Mexico City (Mexico). We performed a retrospective review of data from the Gynaecology Outpatients Department database that included all women referred to the gynaecology service in 2023. Data from a total of 2035 women were screened for eligibility. Consecutive women who fulfilled the following criteria were selected: aged 18 years or older, had a valid HPV test report, had self-reported never having received an HPV vaccine, and had answered the complete questionnaire with sociodemographic data. Women younger than 18 years old who had been previously vaccinated against HPV, did not have an HPV test, or who did not return a valid HPV test result, as well as those with incomplete sociodemographic and clinic data, were excluded. A total of 347 women were selected. The study was conducted in accordance with the Declaration of Helsinki (as revised in 2013) and received ethical approval from the Institutional Review Board and Ethics Committee of INPer (INPer Reg. 2025-1-22). Informed consent was waived due to the retrospective nature of the study. Data were anonymised to ensure patient confidentiality. All methods were performed in accordance with institutional guidelines and regulations.

### 2.2. Specimen Collection and DNA Isolation

All women underwent speculum examination, and cervical samples were obtained using sterile cytobrushes (Steril Medical Co., Ltd., Yangon, Myanmar). Brushes were placed in a tube containing ThinPrep PreservCyt (Marlborough, MA, USA), and samples were stored at −20 °C until processing. A volume of 1 mL of each sample was aliquoted into sterile tubes and centrifuged at 12,000× *g* for 10 min. Cell pellets were washed with sterile phosphate-buffered saline. The DNA was extracted using the NucleoSpin Tissue Kit for DNA from cells and tissue (MACHEREY-NAGEL, Düren, Germany) according to the manufacturer’s instructions. DNA samples were stored at −20 °C until further use.

### 2.3. HPV Detection and Genotyping

HPV was detected and genotyped using the CLART HPV4 (GENOMICA, Madrid, Spain) system, which amplifies an L1 gene fragment by polymerase chain reaction with biotinylated primers. CLART HPV4 allows detection of 35 HPV genotypes (6, 11, 16, 18, 26, 31, 33, 35, 39, 40, 42, 43, 44, 45, 51, 52, 53, 54, 56, 58, 59, 61, 62, 66, 68, 70, 71, 72, 73, 81, 82, 83, 84, 85, and 89). Co-amplification of the human-spiked Cystic Fibrosis Transmembrane conductance Regulator (CFTR) gene was included as a sample quality and process control. Amplicons were detected after hybridisation onto 8-well strips containing DNA-specific probes in a low-density microarray. The results were visualised using an automatic Clinical Array Reader (GENOMICA, Madrid, Spain). Samples that tested negative for CFTR amplification were retested, and the second result was considered conclusive.

### 2.4. Statistical Analysis

Data were imported into an Excel spreadsheet (Microsoft Office) and stratified by HPV status (positive or negative). Data were analysed using the GraphPad Prism 7 software (GraphPad, San Diego, CA, USA). We analysed differences in demographic and clinical factors between groups using the Chi-square test for categorical variables and the Mann–Whitney test for continuous variables. Two-tailed *p* values < 0.05 were considered statistically significant. To address the collinearity of continuous variables, we calculated the variance inflation factor (VIF) for the number of sexual partners, age at sexual debut, age, education level, number of pregnancies, reason for hospital admission at the time of sampling, number of genotypes identified, and whether there was a diagnosis of HPV-associated cervical lesions (low-grade squamous intraepithelial lesion (LSIL), high-grade squamous intraepithelial lesion (HSIL), and cervical cancer). Reason for hospital admission at the time of sampling and diagnosis of HPV-associated cervical lesions had the highest VIF values (1.7 and 1.8, respectively); therefore, we excluded them because they showed higher collinearity. The remaining variables had VIF values around 1, which is the accepted threshold for assuming the absence of collinearity. Therefore, we included them in our model as independent variables. To assess the effect that numeric and categorical variables had on the presence of HPV, we considered a multivariable logistic regression model using Python version 3.11.5 and StatsModel 0.14.0. Our model considered VPH_presence ~ Age_sex_D + Age + Sex_partners + Contraception + Other comorbidities (Diabetes Mellitus II, infertility, Insulin resistance or gestational diabetes). Finally, to assess the effect of the number of genotypes on HSIL, LSIL, or cervical cancer, we used a multivariable logistic regression model considering any HPV-associated cervical pathology diagnosis as the dependent variable and age at sexual debut, number of sex partners, and total number of genotypes detected as independent variables. All of them had VIF values below 1.08.

## 3. Results

### 3.1. Demographic and Clinical Characteristics of the Study Population

The present study included 347 women, 124 (35.73%) of whom were positive for HPV infection, while 223 (64.27%) were negative for HPV. Demographic characteristics and risk factors for HPV infection were similar between the infected and uninfected women (Table 1). Comparing the two groups, the mean age, the mean age at sexual debut, and the mean number of pregnancies were similar in both groups. Although the HPV-infected women were more likely to have more lifetime sexual partners (4.45 versus 3.1), the difference was not statistically significant. The use of contraceptive methods was similar between the infected and uninfected women (Table 1).

Sexually transmitted diseases (STDs), cervicitis, and metabolic disorders were the most frequently detected comorbidities in the studied population. Thus, we evaluated their potential association with HPV infection. Cervicitis; STDs including gonorrhoea, HIV, genital herpes, trichomonas, chlamydia, and syphilis; and metabolic disorders such as diabetes mellitus type 2, insulin resistance, and gestational diabetes were diagnosed in the same proportion in HPV-infected and uninfected women (Table 1).

Furthermore, as mentioned in the methodology, VIF was calculated for each variable to assess collinearity between continuous variables. This analysis showed that HPV-associated cervical lesion diagnosis (LSIL, HSIL, and cervical cancer) and reason for hospital admission at the time of sampling exhibited the highest VIF values (indicating collinearity), leading to the decision to use the HPV-associated cervical lesion diagnosis variable as the dependent variable in subsequent statistical analyses.

Subsequently, a multivariable logistic regression was performed to analyse whether any of the variables were correlated with the presence of HPV. It was found that none of the variables correlated with the presence of HPV, except for “age at sexual debut” and “number of sexual partners” (Table 2), thereby demonstrating the consistency of the data and ruling out other potential confounding factors for our study.

Since the presence of HPV was associated with a diagnosis of LSIL, HSIL, and cervical cancer, we decided to conduct a further linear regression analysis to determine whether the number of HPV genotypes detected in a patient correlates with the occurrence of any of these diagnoses; the results are presented in Table 3. We found a significant relationship between the number of HPV genotypes detected and the occurrence of LSIL, HSIL, or cervical cancer, grouped as the “HPV-associated cervical lesion” condition. Our analysis has revealed that the presence of one or more HPV genotypes, either high- or low-risk, increases the probability of occurrence of LSIL, HSIL, or cervical cancer. The risk of presenting with HPV-associated lesions increases 1.5-fold for each genotype present (OR 1.5, 2 CI 97.5%).

### 3.2. HPV Genotype Distribution in Unvaccinated Women

Overall, 124 women were positive for HPV. We detected HR-HPV genotypes in 92 (74.19%) women, LR-HPV genotypes in 50 (40.32%) women, and pHR-HPV genotypes in 26 (20.96%) women. Overall, 11 HR-HPV genotypes were detected; ranked by prevalence, HPV59 was the most frequent, followed by HPV16, HPV39, and HPV31 (Figure 1). Fifteen LR-HPV genotypes were identified; among them, HPV42 and HPV54 were the most frequently detected, followed by HPV61, HPV62, HPV81, and HPV84, which were detected with the same frequency (Figure 1). In this study, we identified only five pHR-HPV genotypes, with HPV66 being the most prevalent (Figure 1).

Among the HPV-positive women, 72 (58.06%) were infected with a single HPV genotype, while infections with multiple genotypes were detected in 52 (41.94%) cases. The proportion of each HPV genotype detected in single- and multiple-genotype infections is shown in Table 4. Figure 2 shows that single-genotype infections were predominantly caused by HR-HPV genotypes (70.83%), followed by LR-HPV (16.67%) and pHR-HPV (12.5%) genotypes (Figure 2A). Likewise, most multiple-genotype infections consisted of HR-HPV genotypes (41/52). The most prevalent co-infections involved exclusively HR-HPV and LR-HPV genotypes (21/52), followed by co-infections involving exclusively HR-HPV (9/52) (Figure 2B). Multiple-genotype infections consisted of two to five concurrent HPV genotypes. The proportion of each HPV genotype detected in co-infections is shown in Figure S1. 

### 3.3. HPV Genotype Distribution Among Women with and Without Pathologies Directly Affecting the Genital Tract

Since HPV is primarily a genitally transmitted virus, we wanted to analyse the prevalence of HPV infection in women diagnosed with pathologies directly affecting the genital tract, such as uterine myomatosis, condyloma acuminatum, cervical ectopy, LSIL, HSIL, and cervical cancer—collectively named “genital tract pathologies”—and compare it with the viral prevalence in women with other diagnoses (infertility, high-risk pregnancy, and menopause), which were collectively named “non-genital tract pathologies”. Overall, 171 women were diagnosed with genital tract pathologies; among them, 70 were positive for HPV infection, and 101 were negative. On the other hand, 175 women were included in the group with non-genital tract pathologies. Of them, 54 were positive for HPV, and 121 showed a negative HPV test. As shown in Table 5, HPV infection was significantly more prevalent among the women with genital tract pathologies than in those with non-genital tract pathologies. The analysis of HPV genotypes showed that, in both groups, infection with HR-HPV genotypes was the most prevalent, followed by infection with LR-HPV genotypes. Notably, the prevalence of HR-HPV was similar in women with and without genital tract pathologies (Table 5).

### 3.4. HPV Genotype Distribution Among Women with Non-Genital Tract Pathologies

The women included in the group of non-genital tract pathologies had attended INPer for infertility, high-risk pregnancy, and symptoms associated with menopause. HPV infection was detected in 15 out of 40 women admitted for infertility treatment. Most of the HPV-positive women were infected with HR-HPV genotypes. Eleven HPV genotypes were detected in these women, with HPV56 and HPV59 being the most prevalent. Interestingly, HPV16 was observed in only one patient (Figure 3).

High-risk pregnancy is a leading reason for admission to the INPer. Of the 89 unvaccinated pregnant women included in this study, 30 (33.7%) were infected with any HPV genotype. The proportion of pregnant women infected with HR-HPV was higher than that infected with LR-HPV and pHR-HPV. The most prevalent HPV genotypes detected were HPV59, followed by HPV16 and HPV39 (Figure 3).

Women complaining of menopause-associated symptoms were the group showing the lowest proportion of HPV infection. In fact, only 9 of the 46 unvaccinated menopausal women had a positive HPV test, most of which were infected with HR-HPV genotypes (Figure 3).

### 3.5. HPV Genotype Distribution Among Women with Genital Tract Pathologies

As a group, the women admitted to hospital for genital tract pathologies showed a significantly higher prevalence of HPV infection compared with that in women with non-genital tract pathologies. However, the frequency of viral infection varied across diagnoses. The genital tract pathologies diagnosed were condyloma acuminatum, uterine myomatosis, cervical ectopy, LSIL, HSIL, and cervical cancer. Condyloma acuminatum, uterine myomatosis, and cervical ectopy are considered benign lesions; in contrast, LSIL and HSIL are considered precursor lesions of cervical cancer. Almost all these pathologies are associated with HPV infection, except for uterine myomatosis. However, it must be considered that the samples obtained for HPV analysis were collected from the cervical canal using a cytobrush, so we did not analyse samples from specific lesions (e.g., myomas, condylomas, and ectopic glandular tissue).

Samples from 45 subjects with condyloma acuminatum were analysed; 8 were positive for infection (Table 6). A group of 39 women with uterine myomatosis were studied; 16 of them were positive for HPV. Notably, these women were predominantly infected with LR-HPV (Table 6). A total 9 of the 24 cases with a diagnosis of cervical ectopy were reported as positive for HPV infection. In contrast with the women with condylomas and myomas, the women suffering from cervical ectopy were predominantly infected with HR-HPV genotypes (Table 6).

LSIL was the most common gynaecologic abnormality found in this study (47 women). Of these cases, 22 were HPV-positive; the assay detected 12 HR-HPV, 9 LR-HPV, and 2 pHR-HPV genotypes (Figure 4). Single- (12 cases) and multiple-genotype (10 cases) infections were detected. Notably, HR-HPV genotypes were present in all co-infections; therefore, most women (19/22) were infected with HR-HPV, either as single- or multiple-genotype infections. The most commonly found genotypes were HPV39 and HPV59 (Figure 4). A small number of women were diagnosed with both HSIL and cervical cancer. HPV was detected in 9/9 HSIL women and 6/6 cervical cancer cases. Figure 4 shows that HR-HPV was detected in 9/9 HSIL cases and in 5/6 women with cervical cancer.

Overall, our results showed the presence of 15 different LR-HPV, 11 HR-HPV, and 6 pHR-HPV genotypes, either as single- or multiple-genotype infections, in the unvaccinated population studied. Notably, we observed a low prevalence of HPV6 and HPV11—the LR-HPV genotypes targeted by the vaccines—at 4% and 2%, respectively. In contrast, the non-vaccine-targeted LR-HPV genotypes HPV42, 54, 61, and 81 were more frequently observed. In contrast, 6 of the 11 HR-HPV genotypes identified are targeted by the nonavalent vaccine (Table 7). Our data provide information about the circulating HPV genotypes in the gynaecological and obstetrics population, and support introducing the nonavalent vaccine as a core component of the national HPV prevention programme.

## 4. Discussion

In this study, we investigated HPV infections among unvaccinated women with and without gynaecological pathologies. Overall, the prevalence of HPV infection was 35.73%. Furthermore, 74.19% of the positive women were infected with HR-HPV types. In line with our results, a study evaluating the presence of HPV in women aged 18 to 45 years attending a Mother and Child Centre in Kazakhstan found a prevalence of 35.1% in unvaccinated women [13]. Likewise, a study including 150 unvaccinated women from Paraguay reported a prevalence of 56.7% of any HPV type, and of 52.7% of HR-HPV types [14].

The potential association of HPV infection and infertility in women remains controversial. Here, we found that HPV infection was not linked to infertility. Our observation agrees with data reported in a meta-analysis involving 15,450 women, which showed that infection with any HPV type was not associated with female infertility [15]. However, when infections were categorised by viral type, the authors found that HR-HPV infection was significantly associated with infertility. Although we observed that most HPV-positive women suffering from infertility were infected with HR-HPV types, the small number of women included (a total of 40, 15 of which were HPV-positive) prevented us from developing a proper comparison with previously reported data. To determine whether HR-HPV infection is actually linked with reproductive problems in our population, a higher number of women are currently being analysed.

Extensive data indicate that HPV infection is significantly more prevalent in pregnant women than in age-matched, non-pregnant control women [16,17]. However, studies focusing on women with high-risk pregnancies are rare. Here, we found that 33.7% of women with high-risk pregnancies were positive for HPV infection. High-risk pregnancy is associated with pre-existing conditions such as autoimmune disease, diabetes, hypertension, obesity, and kidney disease, along with pregnancy-related conditions such as gestational diabetes, preeclampsia, and placenta previa. The impact of HPV infection on the evolution and outcome of pregnancy remains controversial [18]. In fact, our results showed that the frequency of HPV infection in high-risk pregnant women did not differ from the overall prevalence detected in the whole study population, suggesting that the level of viral infection detected in pregnant women mirrors that in the unvaccinated women studied, and may not be associated with pregnancy problems.

HPV infection generally declines with age. Accordingly, we found that menopausal women were the group with the lowest level of HPV infection. In this study, we detected that 19.56% of the menopausal women tested positive for HPV. Interestingly, more than half presented with an infection with HR-HPV types. Larger studies have demonstrated that the HPV infection curve shows two clear peaks: the first includes women under 25 years of age, and the second includes women over 55 years of age [19]. Moreover, a deeper analysis demonstrated that HPV infection in younger women is due to new acquisition, whereas positivity in older women is mainly due to persistent infection [20]. The cross-sectional nature of our study prevents us from knowing if the HPV infections detected in the menopausal women were persistent. However, it might be considered that to reduce the risk of developing cervical lesions, a patient infected with HR-HPV genotypes might benefit from timely monitoring by a qualified healthcare provider.

The prevalence of HPV infection in the group of women with pathologies directly affecting the genital tract was the highest detected in this study. The genital tract pathologies detected were condyloma acuminatum, uterine myomatosis, cervical ectopy, LSIL, HSIL, and cervical cancer. Condyloma acuminatum is caused by LR-HPV [21], mainly HPV6 and 11. Here, we found a lack of detectable cervical HPV in the cervical specimens evaluated (only 4/37 women were HPV-positive). These results are explained by the fact that the samples used for HPV detection were not obtained from the condyloma, but from the cervix; thus, they do not reflect the virus present in condylomatous lesions. They also suggest that, although condyloma acuminatum is a common sexually transmitted disease [22], it does not seem to be accompanied by a cervical HPV infection, at least in our population.

Uterine myomatosis is a prevalent benign tumour of the uterus that affects fertility and represents a major cause of hysterectomy in women of reproductive age worldwide [23]. This study included a small number of women with myomatosis (N = 39); however, we observed that 16 of them were positive for HPV infection. Myomatosis is not associated with a viral aetiology, but the involvement of pro-inflammatory mediators in its pathogenesis has been proposed [23]. In a previous study, we reported that HPV infection is linked with an inflammatory response in women with cervical ectopy [24]; thus, it would be interesting to explore whether the inflammatory response, potentially produced by the cervical HPV infection, might contribute to the pro-inflammatory microenvironment associated with myomatosis. To test this hypothesis, a dedicated study of the inflammatory mediator profile in HPV-positive and -negative women suffering from myomatosis would be needed.

Cervical ectopy is a benign condition, frequently found during routine pelvic examinations in women of reproductive age. However, a proportion of women experience symptoms attributed to cervical ectopy, such as increased mucus production, pelvic pain, and postcoital bleeding. We previously reported a high prevalence of HR-HPV genotype infection in women with symptomatic cervical ectopy [25]. Here, a limited number of women with cervical ectopy were included (N = 19); five of them were positive for the presence of HPV. Interestingly, four out of the five genotypes detected were HR-HPV types. However, there is no evidence linking the presence of HR-HPV and the development of HPV-associated lesions in women with cervical ectopy; thus, to understand the potential relevance of our observations, follow-up studies including a larger number of women with cervical ectopy are needed.

LSIL and HSIL are HPV-associated cervical dysplasias. LSIL is considered a mild lesion, frequently associated with transient HPV infection, while HSIL is a severe dysplasia considered a precancerous lesion, with a significant risk of progressing to cervical cancer. Accordingly, in this study, we observed that 22/47 LSIL cases were HPV-positive, whereas 9/9 HSIL and 5/6 cervical cancer cases were positive for HR-HPV. Our data agree with previous studies. In one of these studies, the authors analysed 1279 unvaccinated women aged 19 to 83 years, finding that 73.7% of LSIL cases and 96.6% of HSIL cases were positive for HPV. The authors reported that HPV16/18 were the most prevalent types in HSIL [26]. In addition, Kovacevic and colleagues studied 1495 unvaccinated women aged 18 to 65 years. They detected HPV in 70% of LSIL and 82.9% of HSIL cases. A proportion of 93.7% of LSIL and 100% of HSIL cases were infected with HR-HPV [27]. Finally, we found that 6/6 women with cervical cancer were positive for HPV. In line with previously reported data [28], we found that HPV16 was the most commonly detected in these cases, followed by HPV18, 39, and 61. Our data showed that 5/6 cervical cancer cases were attributable to HPV types targeted by the nonavalent vaccine. Since a very small number of women with LSIL, HSIL, and cervical cancer were included in the present study, our results must be interpreted cautiously and should be considered as hypothesis-generating data. It is important to highlight that within our HPV-infected population, nearly half (52/124) presented co-infection with two to five genotypes; our statistical analyses revealed that the presence of multiple genotypes is significantly associated with diagnoses of genital tract pathologies (LSIL, HSIL, and cervical cancer).

An awareness of HPV type distribution is essential for assessing vaccine efficacy and planning rational vaccination programmes for HPV infection prevention. However, updated information about the circulating HPV genotypes in unvaccinated women in Mexico is still scarce. A recent, comprehensive study of the HPV prevalence in 596,944 Mexican women reported an overall prevalence of 37.98% in a subgroup of unvaccinated women (N = 36,865) [29]. Unfortunately, this analysis only included women under 25 years of age. In addition, the HPV genotypes were analysed using a multiplex assay that detects HPV16 and HPV18 as independent results, along with a 12-HR-HPV POOL (HPV31, 33, 35, 39, 45, 51, 52, 56, 58, 59, 66, and 68); thus, although they found that most of the unvaccinated HPV-infected women were positive for genotypes included in the POOL, it would not be pertinent to compare their results with ours because we included women with a wider age range, and detected each genotype individually. In an independent study, Pedroza-Gonzalez et al. [30] analysed a cohort of female university students, 89 of whom declared to be unvaccinated; an overall HPV prevalence of 74.1% was reported. This is much higher than the frequency observed in our study; this might be due to the age of the students included in the prior study (mean age 21.75 years), as younger age is associated with higher HPV infection prevalence [19]. Consistent with our results, the authors reported a high prevalence of HR-HPV genotypes, finding that HPV18, HPV31, and HPV16, which are targeted by the nonavalent vaccine, were the most prevalent genotypes.

This study has several limitations. It was an observational, cross-sectional study, conducted retrospectively; thus, it relied on pre-existing datasets that were not designed for a particular study, may have included confounding variables, and prevented us from detecting relevant data, such as HPV persistence, in the infected women. The study was conducted in a single tertiary-care hospital; thus, it is limited to a selected group of women needing gynaecological and obstetric treatment, which is not representative of the whole Mexican population. Given that the aim of the study was to study unvaccinated women, the number of participants was small. Similarly, the number of women in each pathological subgroup was too small to provide conclusive results. A relevant limitation was that the vaccine status was self-reported by each participant; thus, the study was susceptible to recall bias. In addition, the lack of a control vaccinated population prevented us from ascertaining vaccine effectiveness and providing more supportive information to population health decision makers.

Our findings, though limited due to the small number of women included, provide information about the HPV types circulating in unvaccinated women and suggest that incorporation of the nonavalent vaccine into national HPV prevention programmes, together with proper screening and timely treatment of precursor lesions, may help protect the population against the majority of circulating, cancer-associated HPV genotypes.

## 5. Conclusions

In conclusion, we have shown that a high proportion of unvaccinated women attending a gynaecology and obstetrics hospital are infected with HPV and that oncogenic HPV types are the most prevalent in single- and multiple-HPV type infections. Although limited by the small number of women analysed, our data provide valuable information about the circulating HPV genotypes in unvaccinated women and show that most of the more frequently detected HPV genotypes are targeted by the nonavalent HPV vaccine. These results have clear implications for implementing a rational HPV prevention programme in Mexico, where vaccination coverage remains suboptimal.

## Figures and Tables

**Figure 1:**
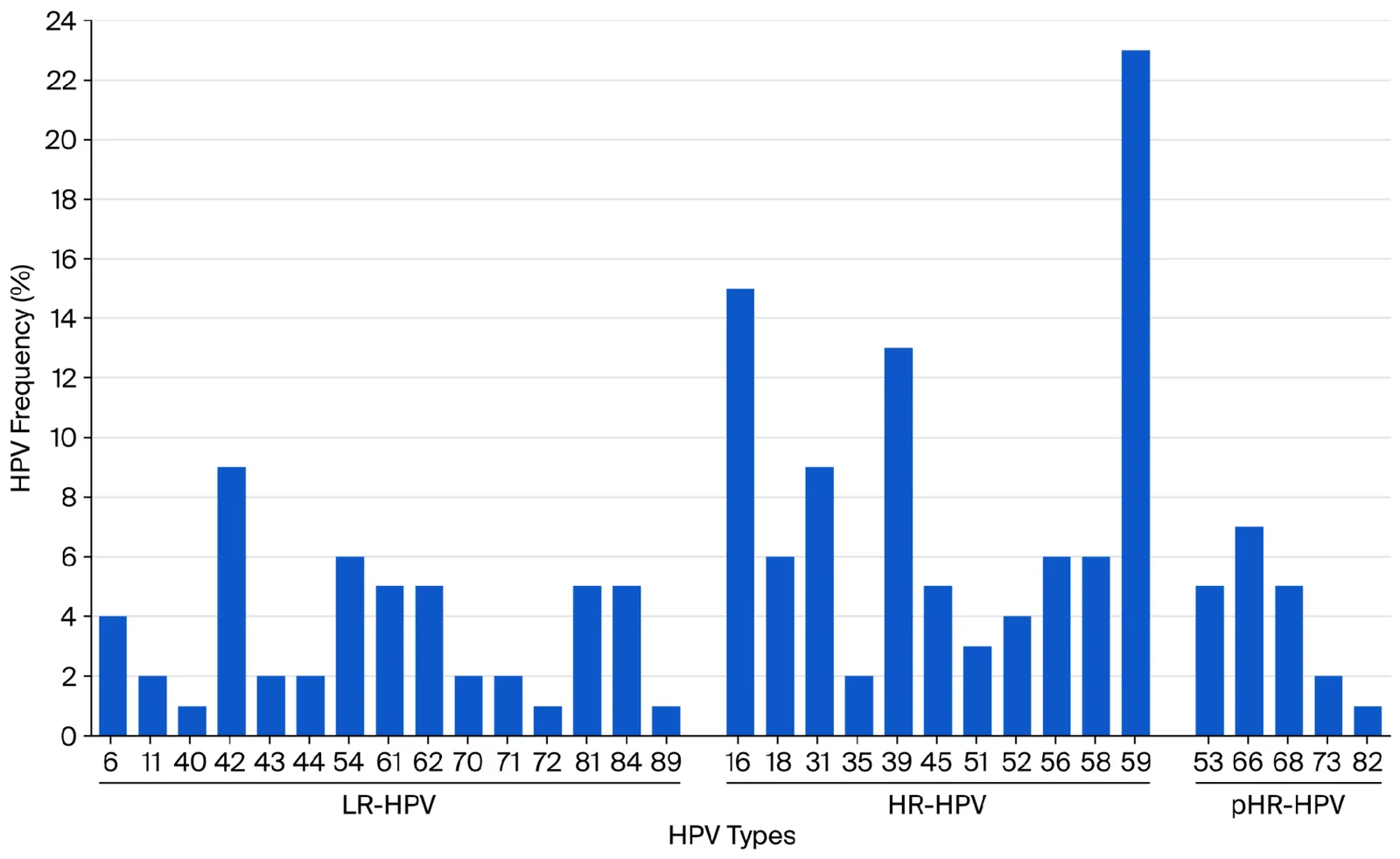
Distribution of HPV genotypes in unvaccinated women. Overall frequency of HPV genotypes among HPV-positive women (N = 124). HPV genotypes were classified according to the IARC carcinogen classification [1]: HR-HPV—high-risk HPV (Group 1, carcinogenic to humans); pHR-HPV—probable and possibly high-risk HPV (Groups 2A and 2B, probably and possibly carcinogenic to humans, respectively); LR-HPV—low-risk HPV (non-carcinogenic to humans). Each genotype includes single- and multiple-genotype infections.

**Figure 2:**
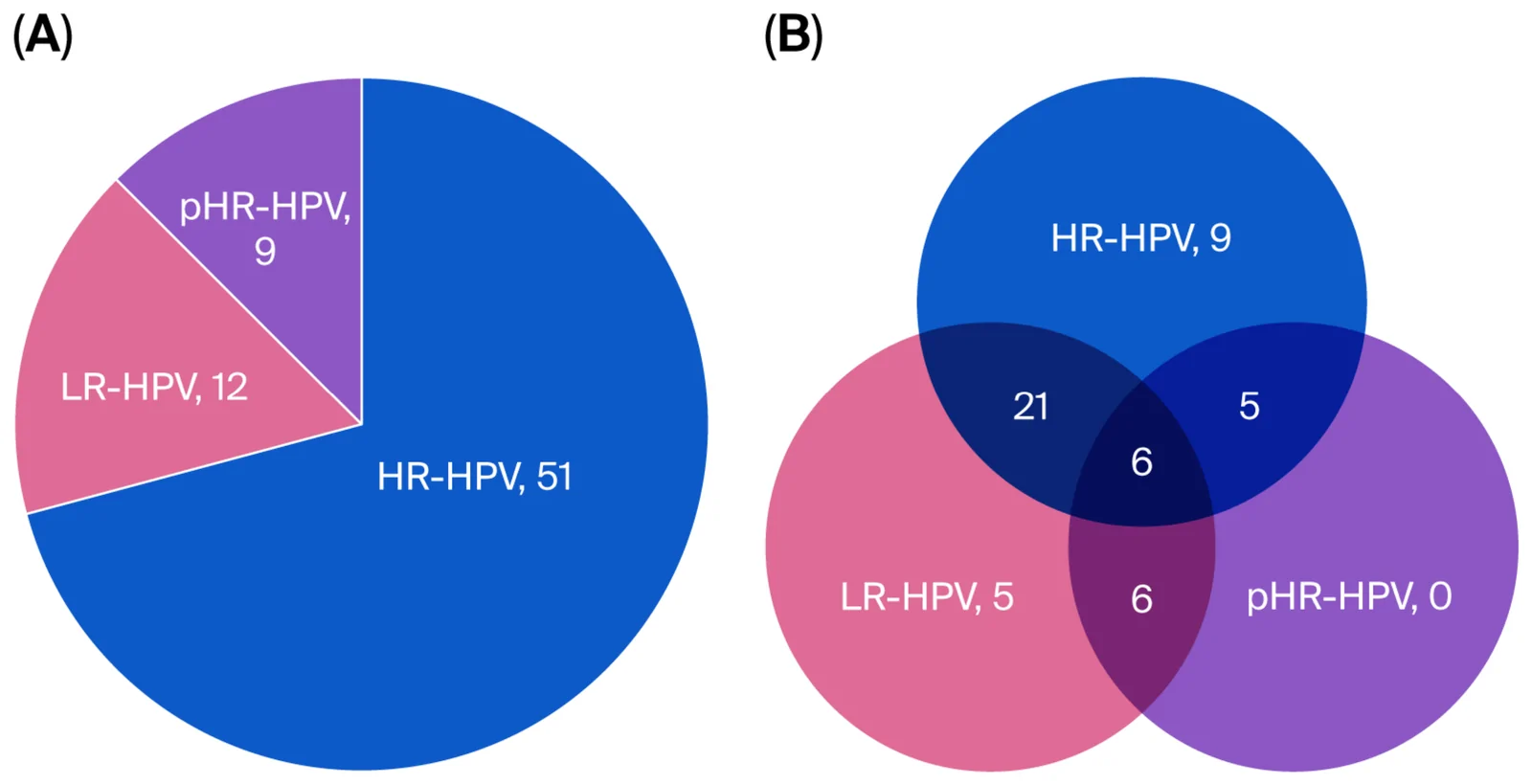
Distribution of HR-HPV—high-risk HPV (Group 1, carcinogenic to humans); pHR-HPV—probable and possibly high-risk HPV (Groups 2A and 2B, probably and possibly carcinogenic to humans, respectively); LR-HPV—low-risk HPV (non-carcinogenic to humans) genotypes in single—(**A**) and multiple-genotype HPV infections (**B**).

**Figure 3:**
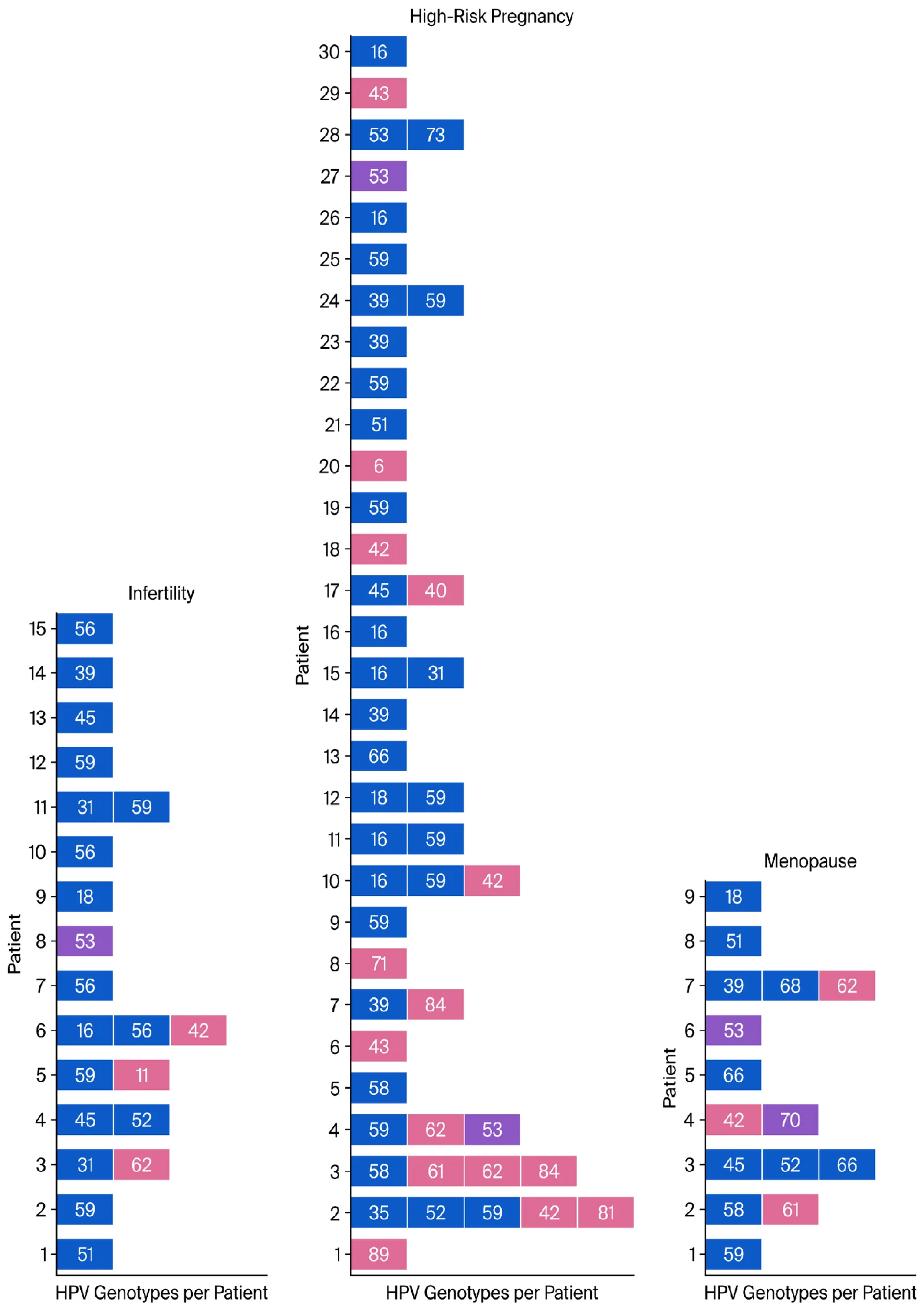
HPV genotypes detected in unvaccinated women admitted to hospital due to infertility, high-risk pregnancy, or menopause-associated symptoms. Blue boxes: HR-HPV—high-risk HPV (Group 1, carcinogenic to humans) genotypes. Pink boxes: LR-HPV—low-risk HPV (non-carcinogenic to humans) genotypes. Violet boxes: pHR-HPV—probable and possibly high-risk HPV (Groups 2A and 2B, probably and possibly carcinogenic to humans, respectively) genotypes.

**Figure 4:**
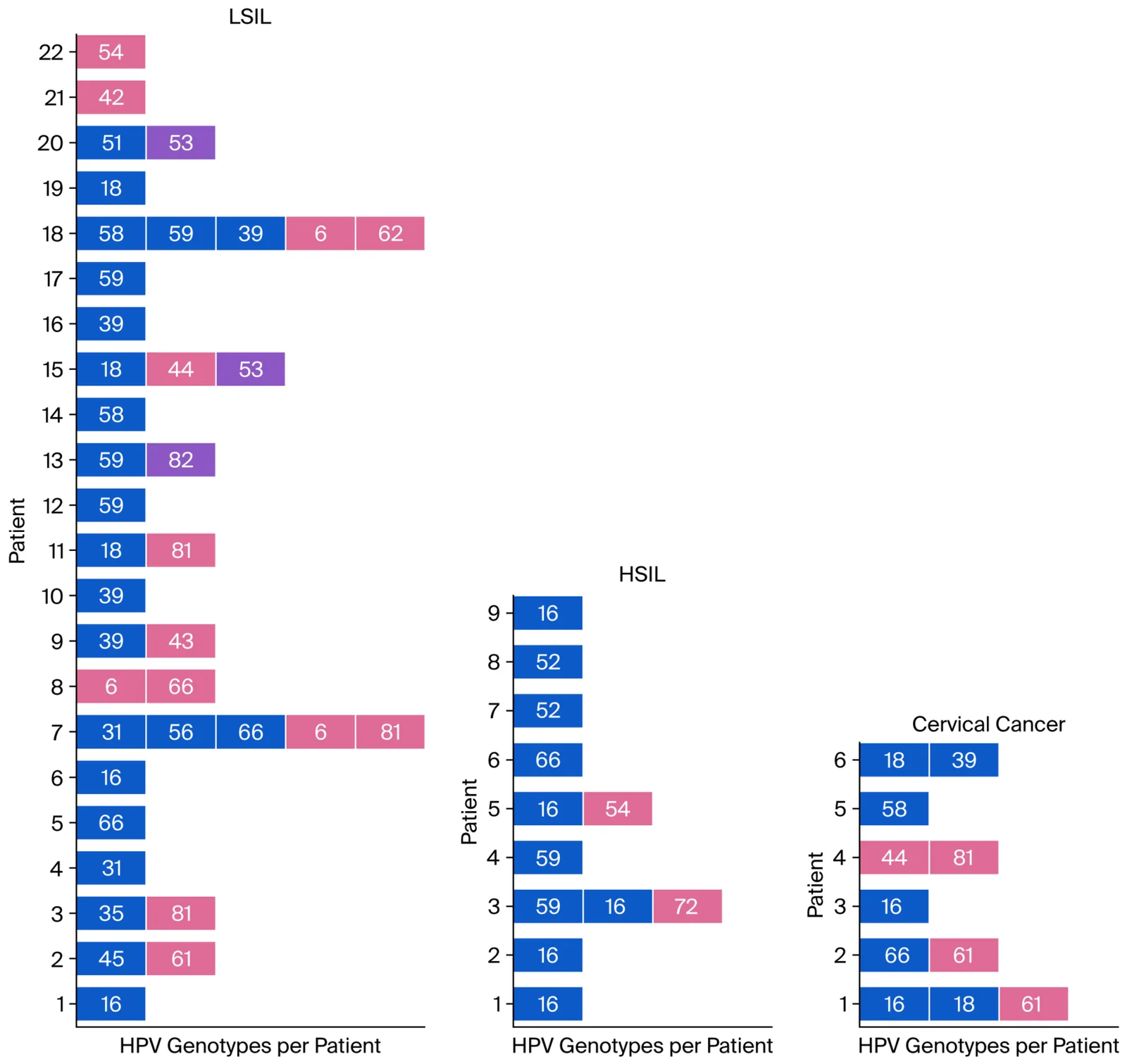
HPV genotypes detected in unvaccinated women diagnosed with low-grade squamous intraepithelial lesion (LSIL), high-grade squamous intraepithelial lesion (HSIL), or cervical cancer. Blue boxes: HR-HPV—high-risk HPV (Group 1, carcinogenic to humans) genotypes. Pink boxes: LR-HPV—low-risk HPV (non-carcinogenic to humans) genotypes. Violet boxes: pHR-HPV—probable and possibly high-risk HPV (Groups 2A and 2B, probably and possibly carcinogenic to humans, respectively) genotypes.

**Table 1:** Demographic and clinical characteristics of HPV-positive and -negative women.

	HPV-Positive (N = 125)	HPV-Negative (N = 222)	*p* Value
Age	39.28 (26–76/IQR: 13.25)	41.43 (18–77/IQR: 14.50)	0.418
Age at sexual debut	18.10 (13–34/IQR: 3.5)	19.41 (13–33/IQR: 4.0)	0.427
Number of sexual partners	4.45 (1–20/IQR: 3.5)	3.19 (1–24/IQR: 2.0)	0.635
Number of pregnancies	2.41 (0–14/IQR: 2.0)	2.43 (0–8/IQR: 3.0)	0.296
Contraceptive use			
Hormonal	4 (3.20%)	11 (4.95%)	0.586
Other	84 (67.20%)	160 (72.07%)	0.460
None	37 (29.60%)	51 (22.97%)	0.245
STD	6 (4.83%)	14 (6.30%)	0.639
Cervicitis	7 (5.6%)	27 (12.6%)	0.059
Type 2 diabetes mellitus	10 (8.0%)	16 (7.2%)	0.832
Insulin resistance	3 (2.4%)	16 (7.2%)	0.083
Gestational diabetes	4 (3.2%)	1 (0.45%)	0.057

A *p* value < 0.05 was considered significant.

**Table 2:** Correlation of HPV infection and sociodemographic and clinical variables, based on multivariable logistic regression analysis.

	Odds Ratio (OR)	C.I. 2.5%	C.I. 97.5%	*p* Value
Age	1.0031 × 10^0^	0.9727	1.0344	0.8461
Age at sexual debut	8.9310 × 10^−1^	0.8205	0.9722	0.0090
Number of sexual partners	1.1314 × 10^0^	1.0284	1.2447	0.0112
Insulin resistance	3.7450 × 10^−1^	0.1006	1.3938	0.1430
Gestational diabetes	9.1372 × 10^0^	0.9201	90.7372	0.0589
Cervicitis	7.8260 × 10^−1^	0.3017	2.0297	0.6141
Diabetes mellitus II	9.0280 × 10^−1^	0.2964	2.7501	0.8572
Pregnancy	1.9387 × 10^0^	0.2495	15.0627	0.5268
Menopause	1.2954 × 10^0^	0.1094	15.3431	0.8374
Uterine myomatosis	3.1839 × 10^0^	0.3611	28.0734	0.2971
Cervical ectopy	3.9043 × 10^0^	0.3975	38.3467	0.2426
Condyloma acuminatum	1.0620 × 10^0^	0.1223	9.2248	0.9565
Infertility	2.9787 × 10^0^	0.3432	25.8489	0.3222
LSIL	5.3391 × 10^0^	0.6015	47.3891	0.1327
HSIL	3.7041 × 10^9^	0.0000	inf *	0.9986
Cervical cancer	1.5486 × 10^12^	0.0000	inf *	0.9999

Abbreviations: Confidence interval, C.I. A *p* value < 0.05 was considered significant * The intercept of the analysis was removed because the variables HSIL and cervical cancer showed collinearity. Hence, the coefficients directly represent the log-odds for the specific category, and the C.I. is infinite (inf).

**Table 3:** Correlation of the presence of HPV-associated cervical lesions (LSIL, HSIL, and cervical cancer) and age, sexual behaviour, and number of HPV genotypes, based on multivariable logistic regression analysis.

	Odds Ratio (OR)	C.I. 2.5%	C.I. 97.5%	*p* Value
Age	0.9831	0.9524	1.0149	0.2939
Age at sexual debut	1.0445	0.9560	1.1412	0.3350
Number of sexual partners	1.0095	0.9167	1.1117	0.8478
Number of genotypes	1.5665	1.2006	2.0440	0.0009

Abbreviations: Confidence interval, C.I. A *p* value < 0.05 was considered significant.

**Table 4:** Prevalence of each HPV genotype detected in single- and multiple-genotype infections.

HPV Genotype	Classification	Number of Infected Women (%)	Number of Women with Single-Genotype Infection	Number of Women with Multiple-Genotype Infection
59	HR	29 (23.28)	14	15
16	HR	18 (14.51)	9	9
39	HR	16 (12.90)	7	9
42	LR	11 (8.87)	4	7
31	HR	11 (8.87)	4	7
66	pHR	9 (7.25)	4	5
54	LR	8 (6.45)	3	5
58	HR	7 (5.64)	3	4
18	HR	7 (5.64)	3	4
56	HR	7 (5.64)	4	3
53	pHR	6 (4.83)	3	3
68	pHR	6 (4.83)	1	5
61	LR	6 (4.83)	0	6
81	LR	6 (4.83)	0	6
84	LR	6 (4.83)	0	6
62	LR	6 (4.83)	0	6
45	HR	6 (4.83)	2	4
52	HR	5 (4.03)	2	3
6	LR	5 (4.03)	1	4
51	HR	4 (3.22)	3	1
35	HR	3 (2.41)	1	2
73	pHR	3 (2.41)	1	2
43	LR	3 (2.41)	2	1
44	LR	2 (1.61)	0	2
71	LR	2 (1.61)	1	1
70	LR	2 (1.61)	0	2
11	LR	2 (1.61)	0	2
40	LR	1 (0.80)	0	1
72	LR	1 (0.80)	0	1
89	LR	1 (0.80)	1	0
82	pHR	1 (0.80)	0	1

**Table 5:** HPV infection and genotype distribution in women with and without pathologies directly affecting the genital tract.

HPV Status	Genital Tract Pathologies * N = 171 N (%)	Non-Genital Tract Pathologies ** N = 175 N (%)	*p* Value
Any HPV genotype	70 (40.93)	54 (30.85)	0.043
HR-HPV *	43 (25.15)	46 (26.28)	0.908
LR-HPV ^#^	29 (16.95)	23 (13.14)	0.404
pHR-HPV ^#^	5 (2.92)	10 (5.71)	0.218

HPV: human papillomavirus. HR-HPV: high-risk HPV (Group 1, carcinogenic to humans); pHR-HPV: probable and possibly high-risk HPV (Groups 2A and 2B, probably and possibly carcinogenic to humans, respectively); LR-HPV: low-risk HPV (non-carcinogenic to humans). * Genital tract pathologies: uterine myomatosis, condyloma acuminatum, cervical ectopy, LSIL, HSIL, and cervical cancer). ** Non-genital tract pathologies: infertility, high-risk pregnancy and menopause. ^#^ Data include detection of genotypes in single- and multiple-genotype infections.

**Table 6:** HPV genotype distribution in women diagnosed with condyloma acuminatum, uterine myomatosis, and cervical ectopy.

Diagnosis	Patient	HPV Genotype
Condyloma acuminatum	1	31
	2	54
	3	54
	4	59, 84
	5	16, 42, 59
	6	11, 59
	7	39
	8	39, 59
Uterine myomatosis	1	6, 54
	2	31
	3	31, 68
	4	31, 84
	5	42, 54
	6	59
	7	59
	8	61, 70
	9	68
	10	39
	11	68, 73, 84
	12	73
	13	42
	14	45
	15	39, 84
	16	59
Cervical ectopy	1	31
	2	39
	3	42
	4	54, 62
	5	56
	6	39, 56, 68, 42
	7	45, 81
	8	59
	9	31, 35, 66

**Table 7:** Targeting of the HPV genotypes detected by the quadrivalent and nonavalent HPV vaccines.

HPV Genotype	HPV Carcinogenic Classification	Targeted by Quadrivalent Vaccine	Targeted by Nonavalent Vaccine
16	HR	YES	YES
18	HR	YES	YES
31	HR	NO	YES
35	HR	NO	NO
39	HR	NO	NO
45	HR	NO	YES
51	HR	NO	NO
52	HR	NO	YES
56	HR	NO	NO
58	HR	NO	YES
59	HR	NO	NO
6	LR	YES	YES
11	LR	YES	YES
40	LR	NO	NO
42	LR	NO	NO
43	LR	NO	NO
44	LR	NO	NO
54	LR	NO	NO
61	LR	NO	NO
62	LR	NO	NO
70	LR	NO	NO
71	LR	NO	NO
72	LR	NO	NO
81	LR	NO	NO
84	LR	NO	NO
89	LR	NO	NO
53	pHR	NO	NO
66	pHR	NO	NO
68	pHR	NO	NO
73	pHR	NO	NO
82	pHR	NO	NO

HR: high-risk (Group 1, carcinogenic to humans) genotypes. LR: low-risk (non-carcinogenic to humans). pHR-HPV: probable and possibly high-risk (Groups 2A and 2B, probably and possibly carcinogenic to humans, respectively).

## Data Availability

The original contributions presented in this study are included in the article/Supplementary Materials. Further inquiries can be directed to the corresponding authors.

## Supplementary Materials

The following supporting information can be downloaded at: https://www.mdpi.com/article/10.3390/microorganisms14102210/s1, Figure S1: HPV genotype distribution in co-infections.

## Abbreviations

The following abbreviations are used in this manuscript:

CFTR	Human-spiked cystic fibrosis transmembrane conductance regulator
DNA	Deoxyribonucleic acid
HSIL	High-grade squamous intraepithelial lesion
HIV	Human immunodeficiency virus
HPV	Human papillomavirus
HR-HPV	High-risk human papillomavirus
INPer	National Institute of Perinatology
LR-HPV	Low-risk human papillomavirus
LSIL	Low-grade squamous intraepithelial lesion
pHR-HPV	Probably and possibly high-risk human papillomavirus
Pap	Papanicolaou
STDs	Sexually transmitted diseases
