# Supplementary material for: Human Papillomavirus Prevalence and Genotype Distribution in Unvaccinated Women: A Gynaecology and Obstetrics Hospital-Based Retrospective Study in México
Source: Microorganisms. 2026 Oct 1;14(10):2210. doi: 10.3390/microorganisms14102210 (PMC13707723; doi:10.3390/microorganisms14102210)
Supplement: Supplementary file 1 [file microorganisms-14-02210-s001.zip › microorganisms-4520236-supplementary.pdf]

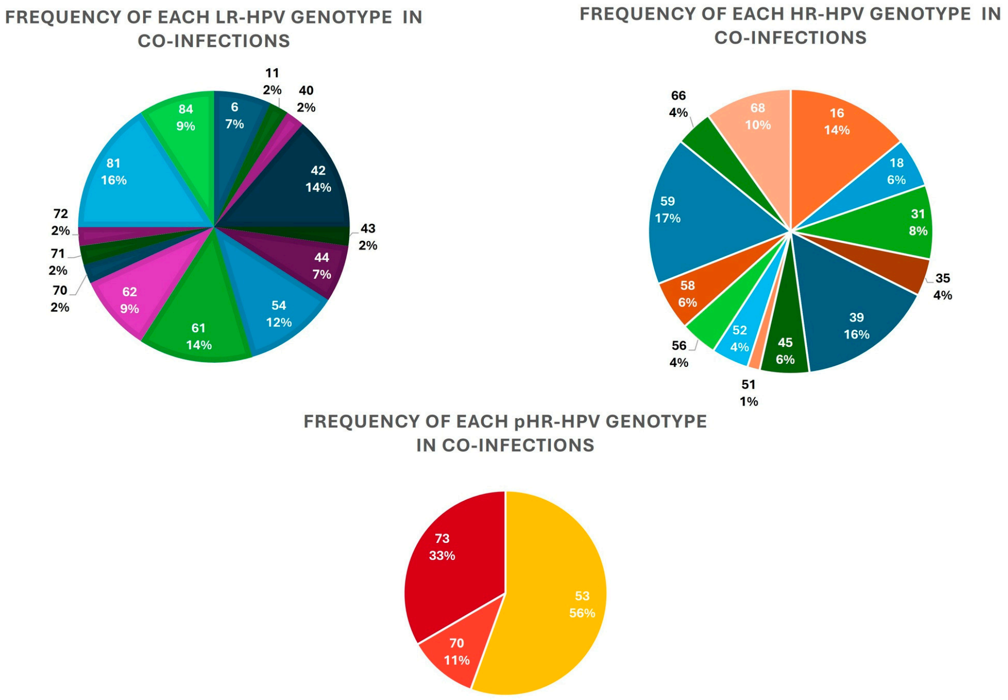

Supplementary Figure S1. HPV genotype distribution in co-infections. Proportion of each low-risk HPV (LR-HPV), high-risk (HR-HPV) and probably high-risk HPV (pHR-HPV) genotype in co-infections. The HPV genotype and its proportion (%) are shown within the pie chart.
